# Diagnostic Accuracy of Methods Used to Detect Cracked Teeth

**DOI:** 10.1002/cre2.70138

**Published:** 2025-04-30

**Authors:** Veronica Kindaro, Huon Molland, Sara Shirbegi, Paul Renner, Unni Krishnan

**Affiliations:** ^1^ School of Dentistry The University of Queensland Brisbane Australia

**Keywords:** accuracy, cracked teeth, diagnostic methods, micro‐CT, transillumination

## Abstract

**Objectives:**

Cracked tooth diagnosis is challenging due to the unknown diagnostic accuracy of tools, resulting in misdiagnosis and suboptimal treatment outcomes. The primary objective of this study was to determine the diagnostic accuracy of four commonly used visual tests in diagnosing cracked teeth based on clinical appearance, independent of patient symptoms. The secondary objective was to assess if clinical experience influences the ability to accurately identify the presence of a crack. The tertiary objective was to assess the ability of the index tests to accurately determine the location of the crack.

**Material and Methods:**

The test sample included 30 teeth extracted due to a suspected crack. Index tests included macrophotography, surgical microscope, transillumination, and DIAGNOcam. Microcomputed tomography (micro‐CT) served as the gold standard. Four examiners of varying experience assessed images of each tooth paired with each index test. The examiner's findings were compared against micro‐CT to determine the diagnostic accuracy of index tests. The relationship between clinical experience and diagnostic accuracy was explored.

**Results:**

Transillumination demonstrated the highest accuracy (65.3%) and sensitivity (68.8%) for diagnosing cracks. Macrophotography and high‐magnification microscope had the highest specificity of 92.9%. Positive predictive value (PPV) was greatest with high‐magnification microscope (96.7%). The low‐magnification microscope demonstrated the lowest accuracy (52.2%). Intra‐rater reliability was moderate to substantial, and inter‐rater reliability was fair. Experienced dentists were more accurate in detecting cracked teeth.

**Conclusions:**

Visual diagnostic methods cannot definitively diagnose cracks. Further studies are required to explore the impact of a combination of tools in diagnosing cracked teeth.

## 1 Introduction

Cracks are ubiquitous in biomineralised structures subjected to loading. Cracks that extend into dentine are potential portals of entry for microorganisms into the dental pulp. Depending on the demographic observed, the prevalence of cracked teeth can range from 10% to 70% (Krell and Rivera 2007). Although the incidence and prevalence of cracks have historically been greater in restored teeth than in unrestored teeth, recent studies have demonstrated that the prevalence of cracks in unrestored teeth has risen (Yap et al. 2023). It has been reported that approximately 46% of cracked teeth present symptomatically, including pain on biting and release, sensitivity to cold, tenderness to percussion and headaches (Krell and Rivera 2007; Hilton et al. 2017; Kakka et al. 2022). However, the symptoms can vary considerably and mimic orofacial pain, headaches and trigeminal autonomic cephalgias (Kakka et al. 2022). These symptoms are not exclusive to cracked teeth and may suggest other dental conditions, including symptomatic pulpitis, apical periodontitis or occlusal trauma, causing diagnostic difficulties (Banerji et al. 2010). A recent survey of Australian dentists confirmed that the primary method used to identify cracked teeth is to test for pain on biting, despite evidence that such symptoms are insufficient to exclude other differential diagnoses (Fong et al. 2023). The same study emphasized that dentists possessed varying diagnostic and treatment preferences when presented with a cracked tooth (Fong et al. 2023). This is possibly due to the lack of an ideal diagnostic tool to detect a crack's presence and depth. No clinically relevant studies have followed the STARD guidelines for testing the diagnostic accuracy of such tools. To address these inconsistencies in diagnosis, the accuracy of different diagnostic tests must be evaluated to detect cracked teeth optimally (Fong et al. 2023; Yu et al. 2022).

Macrophotography is a more affordable alternative to microscopy for clinicians who may have limited access to advanced diagnostic tools, and it has not been evaluated for diagnostic accuracy at crack detection (Ahmad 2009). Macrophotography can produce high‐resolution images that may assist clinicians with visualizing and assessing surfaces of the dentition (Ahmad 2009). They help establish a baseline record of a tooth's clinical appearance for later comparison, analysis, and medico‐legal evidence (Eichenberger et al. 2018).

Magnification has been proven to increase precision and accuracy, particularly when visual clarity is compromised (Behle 2001). 77% of recently surveyed dentists routinely use magnification (Fong et al. 2023). Surgical microscopes provide a standardized way of studying cracks and have adjustable magnifications, significant illumination, and the ability to capture intraoral images (Behle 2001).

Fiber‐optic transillumination has been adapted as a diagnostic tool for cracked teeth (Davies et al. 2001). Transillumination relies on the concept that a disruption in light transmission through a tooth may indicate the presence of a crack (Zidane 2022). 57% of dentists were found to use transillumination when a cracked tooth is suspected (Fong et al. 2023). Although transillumination remains a popular choice for cracked tooth diagnosis, previous studies have shown that it has some inherent disadvantages (Zidane 2022) A reduction in the intensity of transmitted light, as demonstrated by transillumination, does not always suggest the presence of a clinically significant crack. Transillumination can emphasize the presence of craze lines that do not require intervention. In addition, subtle changes in light transmission may be difficult for the operator to interpret (Zidane 2022).

DIAGNOcam (KaVo Dental, Germany) is primarily marketed as a caries detection tool; however, the manufacturer states it can be used for crack detection using the transillumination function (Dental 2024). It uses the projection of near‐infrared light (850 nm) to detect cracks (Dental 2024). As opposed to having a single light source, as seen in fiber‐optic transillumination, DIAGNOcam emits light from two sources in opposite directions. In addition, DIAGNOcam has a multi‐image capture feature that takes three intraoral photos of the tooth: one plain intraoral photo, one with transillumination, and the other with fluorescence. As its fluorescence functionality is primarily for detecting caries, it can be disabled for crack detection (Sridhar et al. 2009). A previous paper with significant limitations only briefly investigated the application of DIAGNOcam as a crack detection tool (Hausdörfer et al. 2023).

For this study, a crack has been defined as a discontinuity in the coronal tooth structure that extends into the dentin. The aim of this project was to investigate the accuracy of macrophotography, a surgical microscope, fiber‐optic transillumination (Microlux 2, AdDent Inc.), and DIAGNOcam in detecting cracks that extend past the dentino‐enamel junction in posterior teeth. The primary objective of this study was to determine the diagnostic accuracy of visual tests used to diagnose cracked teeth based on clinical appearance, independent of patient symptoms. The secondary objective was to assess if clinical experience influences the ability to accurately identify the presence of a crack. The tertiary objective was to assess the ability of the index tests to accurately determine the location of the crack.

## 2 Methodology

### 2.1 Ethics and Sample Collection

A low or negligible risk (LNR) ethics clearance from Human Research Ethics Committees was obtained under project number 2023/HE001646. 30 teeth that were extracted due to suspected cracks were used in this study. The retention period after extraction varied from 3 to 9 months.

### 2.2 Study Design and Setting

This is an ex vivo quantitative diagnostic accuracy study and has been designed with the STARD Guidelines and the QUADAS‐2 quality assessment tool as a guideline to minimize the risk of bias and produce valid data (Cohen et al. 2016; Whiting et al. 2011).

### 2.3 Data Collection

All extracted teeth were initially stored in formalin and later transferred to physiological saline. Care was taken to keep the teeth hydrated after individual index tests, and the teeth were never left out to dry. The individual tooth was placed in silicone putty and mounted in an endodontic sextant. The sextant was placed in a phantom head mounted on a dental chair to simulate patient positioning during dental treatment. All fiber‐optic transillumination and macrophotography index test images were captured using a Nikon D850 camera (Nikon Corporation, Japan) and a Nikon 105 macro lens with a 20 mm extension tube (Kenko, Japan). A CJ‐Optik Flexion Basic Dental Microscope (CJ Optik GmbH & Co, Germany) was used at 0.4×, 0.6×, and 2.5× microscope turret settings, which equated to 5.1× (low‐magnification), 12.5× (medium‐magnification) and 21.25× (high‐magnification) magnifications. The surgical microscope had a focal distance tube of 170 mm, focal distance of lens of 250 mm, and an eyepiece factor of 12.5×. The multi‐image capture setting was used for DIAGNOcam. However, the fluorescence image was not shown to the examiners.

Micro‐CT images of each tooth served as the gold standard. The micro‐CT (Skyscan 1272, Bruker, MA, USA), parameters included an acquisition time of 2.68 min, pixel size of 0.007 mm, 100 kV source voltage, 100 μA source current, and 0.11 mm Cu filter. Each tooth was kept hydrated during the scan. An endodontist assessed the micro‐CT results and was blinded to data acquired from the four examiners. Cracks confined to the enamel were ignored. Samples with crack extension into dentine were considered as true positives.

**Figure 1:**
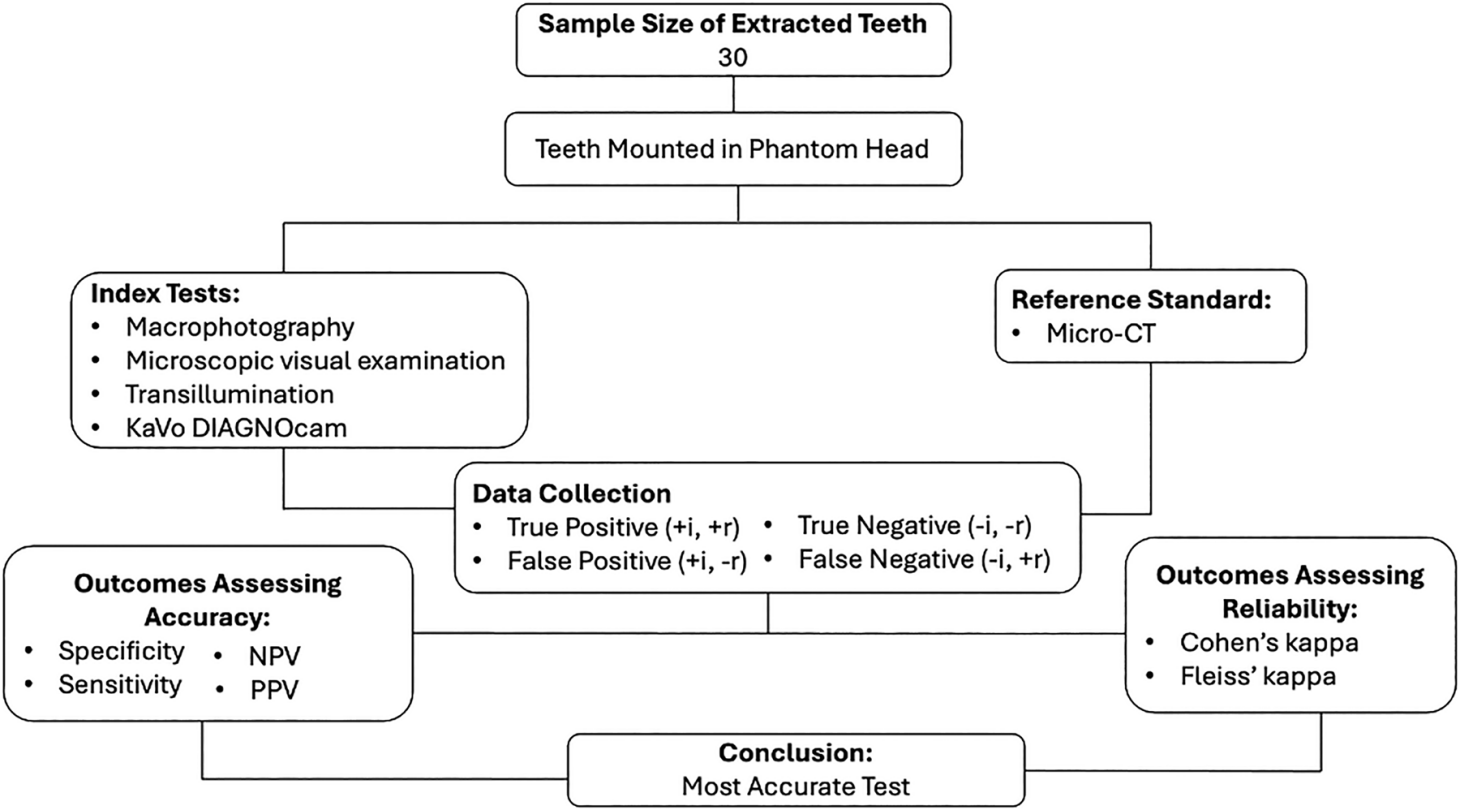
Flow chart of study design.

A pilot study used 20 sample images that an experienced dentist viewed to test the sequencing, methodology, and data collection. Four different examiners were selected for the study, which included two recent dental graduates with less than 5 years of experience and two senior dentists with over 20 years of experience (Figure 1).

**Figure 2:**
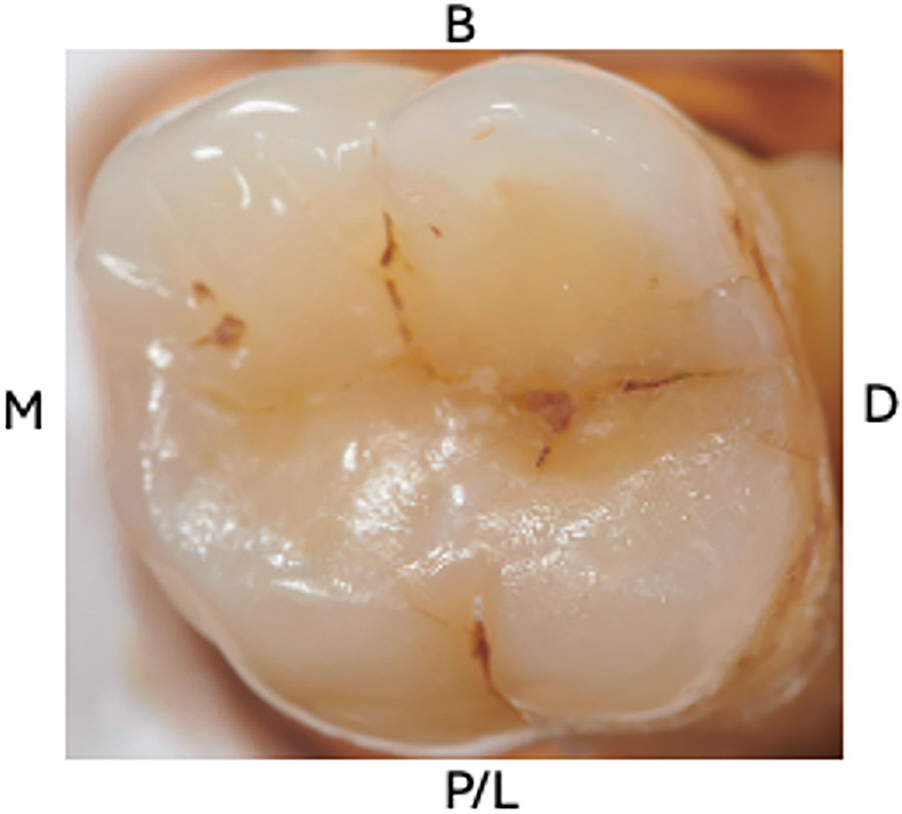
Image of a sample depicting macrophotography as an index test.

**Figure 3:**
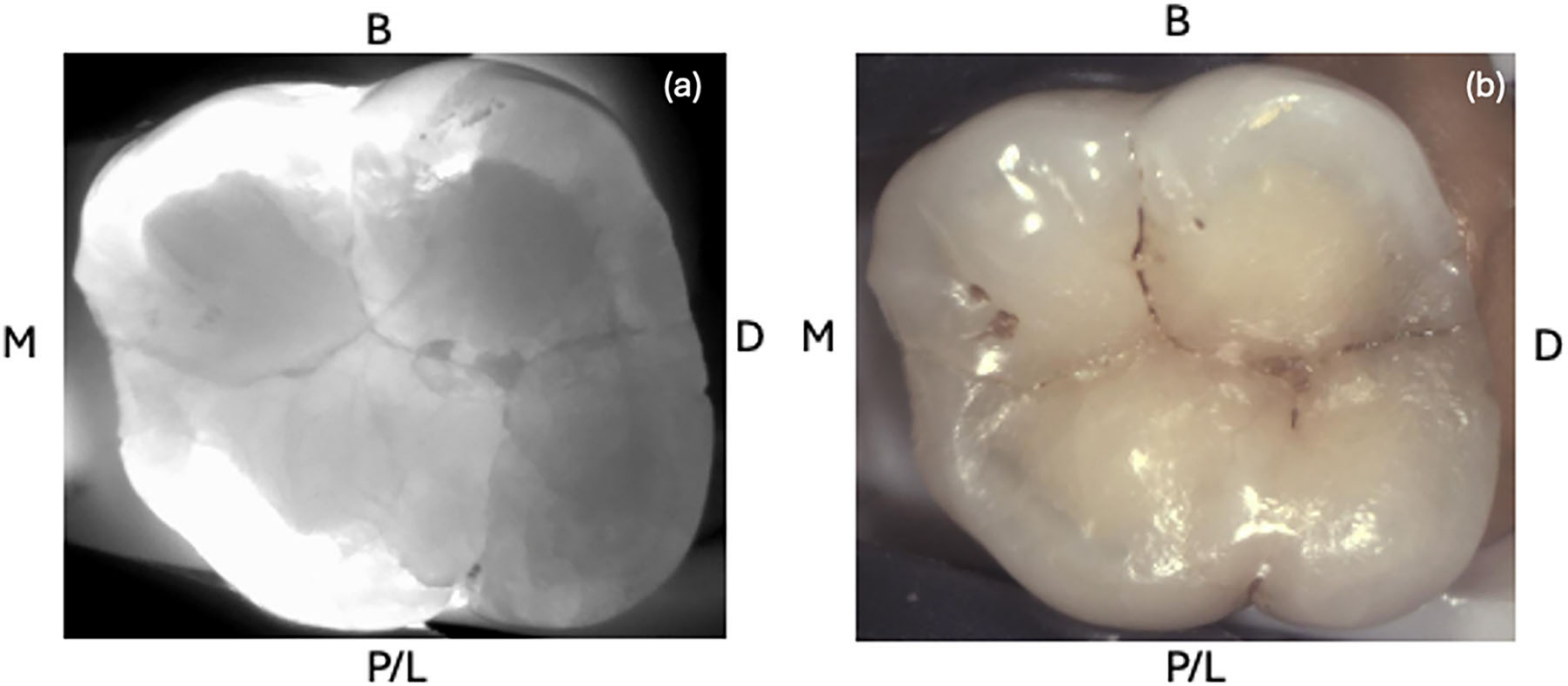
Images of a sample depicting (a) DIAGNOcam as an index test using its transillumination component and (b) DIAGNOcam plain intraoral photo.

**Figure 4:**
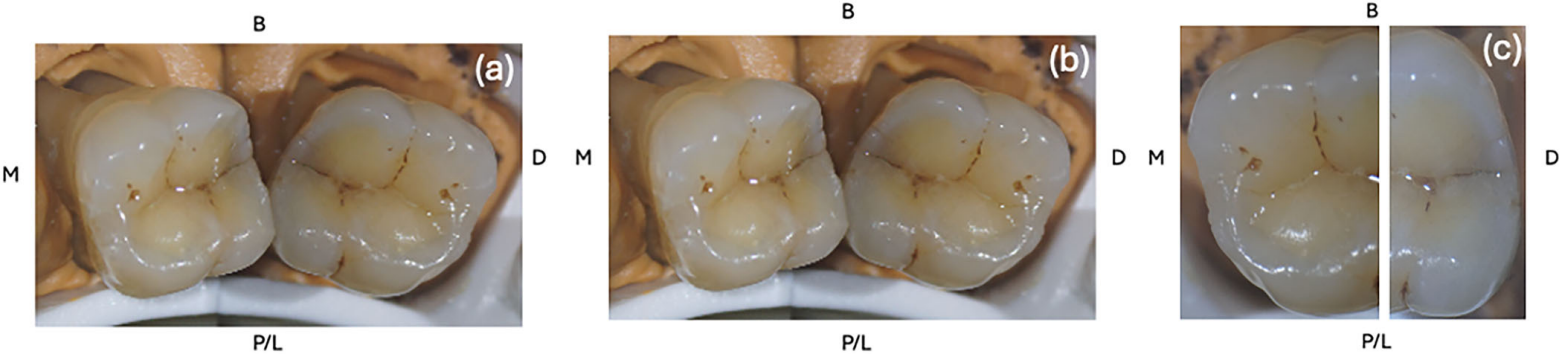
Images of a sample depicting microscope as an index test of various magnifications (a) ×3.4 low‐end magnification, (b) ×5.1 medium‐end magnification, and (c) mesio‐occlusal and disto‐occlusal surface at ×21.25 high‐end magnification.

**Figure 5:**
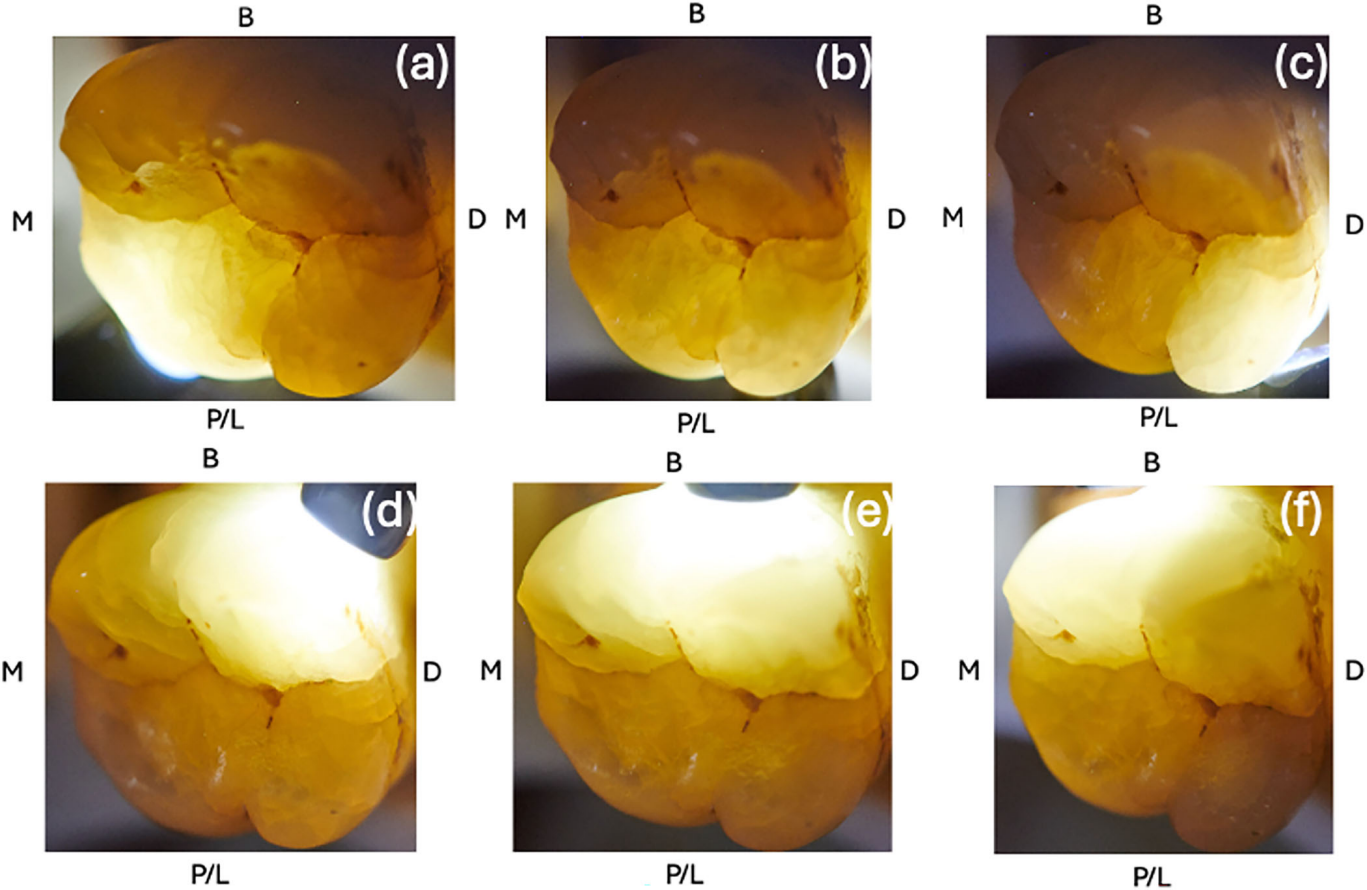
Images of a sample depicting fiber‐optic transillumination from six sites: (a) mesio‐lingual transillumination, (b) mid‐lingual transillumination, (c) disto‐lingual transillumination, (d) Disto‐buccal transillumination, (e) Mid‐buccal transillumination, and (f) Mesio‐buccal transillumination.

As presented in Figures 2, 3, 4 and 5, a total of 13 index test images (one macrophotography, two DIAGNOcam, four microscope images, and six transillumination) were captured of each tooth and compiled into a PowerPoint slideshow (File S1). The photos were randomized to ensure that index test images of the same sample were not shown consecutively. Each slide contained images of a single index test with surfaces labeled accordingly (buccal, palatal/lingual, mesial, and distal). A mirror image was sometimes provided to allow the examiner to better depict the tooth.

Two Zoom sessions scheduled 2 weeks apart were hosted per examiner. PowerPoint slides 1–99 were presented in session one and slides 80–180 in session two to give a 10% overlap to calculate intra‐rater reliability. Kappa statistics were used to calculate intra‐rater and inter‐rater reliability.

Prompts that were given to the examiners included the following:

1. Is there a crack or cracks present in the sample? Yes, No, or Unsure responses were accepted.
2. If there is a crack or cracks, identify the location and annotate them accordingly.
3. Does the crack stop in the enamel, or does it propagate into the dentine?

### 2.4 Data Analysis

Data collected from the four examiners and the micro‐CT were categorized as:

- True positive (TP) – Positive index and positive reference
- True negative (TN) – Negative index and negative reference
- False positive (FP) – Positive index and negative reference
- False negative (FN) – Negative index and positive reference

Enamel cracks and unsure responses identified by the examiners were re‐coded to “no crack.” Once all data was coded, the Jamovi software was used to perform accuracy, specificity, sensitivity, positive predictive value, and negative predictive value calculations.

In summary, sensitivity is the proportion of cracked teeth correctly identified by the index test, and specificity is the proportion of teeth without cracks that are correctly identified (Campbell et al. 2015). PPV is the likelihood that a tooth identified as cracked is truly cracked, whereas NPV is the likelihood that a tooth identified as not cracked is truly not cracked (Campbell et al. 2015). Kappa statistics were also calculated to evaluate the intra‐ and inter‐rater reliability. Fleiss' kappa analysis was conducted to assess agreement across all four examiners. A percentage calculation was performed and averaged across the examiner data to determine the accuracy of the index tests in identifying the location of the cracks. The location‐accuracy percentage was calculated as follows:

$$\text{Location}\;‐\;\text{Accuracy}=\frac{\mathrm{Number}\;\mathrm{of}\;\mathrm{surfaces}\;\mathrm{correctly}\;\mathrm{identified}\;\mathrm{as}\;\mathrm{cracked}\;\mathrm{by}\;\mathrm{examiner}}{\mathrm{Cracks}\;\mathrm{identified}\;\mathrm{by}\;\mathrm{micro}\;‐\;\mathrm{CT}}\;\times 100\%$$

Example calculations have been listed below:

**Example 1:** If micro‐CT demonstrates an MODL crack on a tooth, and an examiner identifies an MO crack on that tooth, then their location accuracy would be 50%.

**Example 2:** If micro‐CT demonstrates an MODL crack on a tooth, and an examiner identifies an MOP crack on that tooth, then their location accuracy would be 50% as two of the four surfaces were correctly identified. The erroneously identified P surface is ignored.

**Example 3:** If micro‐CT demonstrates no crack on a tooth, and an examiner identifies no cracks on that tooth, their location accuracy would be 100%.

## 3 Results

Of the 30 teeth examined under micro‐CT, 80% demonstrated cracks extending past the dentino‐enamel junction. Transillumination demonstrated the highest accuracy and sensitivity for determining cracks at 65.3% and 68.8%, respectively (Table 1). A low‐magnification microscope, however, demonstrated a significantly lower accuracy of 52.2% and a sensitivity of 48.0%. Macrophotography and high‐magnification microscopes had the highest specificity of 92.9%, indicating they were most accurate at excluding the presence of cracks. The high‐magnification microscope had the highest PPV (96.7%), followed by macrophotography (96.4%), indicating that when a positive result was obtained from these index tests, the tooth was highly likely to be cracked. Transillumination was the most accurate, and the low‐magnification microscope was the least accurate in identifying crack location.

**Table 1:** Accuracy of index tests.

Index Test	Accuracy^a^	Sensitivity^a^	Specificity^a^	PPV^a^	NPV^a^	Location Accuracy^b^
Macrophotography	60.6 (51.7, 69.0)	51.9 (41.9, 61.8)	92.9 (76.5, 99.1)	96.4 (87.7, 99.6)	34.2 (23.7, 46.0)	51.0
Microscope						
Low‐Mag Microscope	52.2 (43.5, 60.8)	48.0 (38.0, 58.2)	64.7 (46.5, 80.3)	80.3 (68.2, 89.4)	29.3 (19.4, 41.0)	43.4
Medium‐Mag Microscope	55.1 (46.4, 63.7)	50.0 (40.4, 59.6)	79.2 (57.8, 92.9)	91.8 (81.9, 97.3)	25.3 (16.0, 36.7)	44.4
High‐Mag Microscope	62.5 (53.8, 70.6)	54.6 (44.8, 64.2)	92.9 (76.5, 99.1)	96.7 (88.7, 99.6)	34.7 (24.0, 46.5)	49.9
DIAGNOcam	64.4 (55.6, 72.5)	65.4 (55.4, 74.4)	60.7 (40.6, 78.5)	86.1 (76.5, 92.8)	32.1 (19.9, 46.3)	51.5
Transilluminator	65.3 (56.3, 73.6)	68.8 (58.5, 77.8)	53.6 (33.9, 72.5)	83.5 (73.5, 90.9)	33.3 (20.0, 49.0)	54.6

^a^ Summarized as percentage (95% confidence interval).

^b^ Summarized as percentage.

Cohen's kappa revealed that intra‐rater reliability for Examiners 1, 2, and 3 fell into the moderate category of kappa scores (0.4 < 0.6), whereas Examiner 4 had a substantial (> 0.6) intra‐rater agreement score at 0.65 (Table 2). Fleiss' kappa demonstrated that the overall agreement score between the four examiners is considered fair at 0.275. Cohen's kappa also showed that the senior examiners (Examiners 1 and 2) had a fair agreement. The recently graduated examiners (Examiners 3 and 4) also had a fair agreement (Table 3).

**Table 2:** Intra‐rater reliability assessed over 19 overlapping trials per examiner.

Examiner	Intra‐rater reliability
Examiner 1	74 (0.481, 0.027)
Examiner 2	79 (0.596, 0.005)
Examiner 3	79 (0.513, 0.025)
Examiner 4	84 (0.650, 0.004)

Summarized as percentage (Kappa score, *p*‐value).

**Table 3:** Inter‐rater reliability comparison of two groups of examiners summarized as percentage of agreement (Kappa scare, *p*‐value).

Examiner	Inter‐rater reliability
Senior dentists (1 and 2)	66 (0.306, < 0.001)
New dentists (3 and 4)	61 (0.252, < 0.001)
All dentists (1, 2, 3, and 4)	33 (0.275, ‐)

*Note:* Summarized as percentage (Kappa score, *p*‐value).

Of all examiners, Examiner 4, a new‐graduate dentist, was most accurate at identifying cracks with 70.4% accuracy (Table 4). Examiner 3, the other new‐graduate dentist, demonstrated the lowest accuracy at 43.2%. The average accuracy of experienced dentists, 64.6%, exceeded the 56.8% accuracy of the inexperienced dentists. The chi‐square test result (*p* = 0.011) showed a statistically significant difference between the two groups of examiners.

**Table 4:** Accuracy by examiner at determining presence of crack.

Examiner	Accuracy
a Examiner 1	64.3 (57.2, 71.0)
a Examiner 2	64.8 (57.8, 71.4)
a Examiner 3	43.2 (36.2, 50.4)
a Examiner 4	70.4 (63.5, 76.6)
a Experienced average (1 and 2)	64.6 (59.7, 69.3)
a Inexperienced average (3 and 4)	56.8 (51.8, 61.7)
b Chi‐square test ($\chi^{2}$) (1df)	6.54 (0.011)
b Log odds ratio (Exact)	0.428 (0.013)

^a^ Summarized as percentage (95% CI).

^b^ Summarized as number (*p*‐value).

The raw data from the study was used to extrapolate the usefulness of each diagnostic tool in populations with different prevalences of cracked teeth. To accomplish this, the PPV and NPV were plotted at different prevalence rate of cracks ranging from 0% to 100% (Figure 7). The plot shows that high‐magnification microscope and macrophotography performed better across a wide range of prevalence and low magnification (×5.1) performed the least.

## 4 Discussion

This study explored the accuracy of four standard visual diagnostic tests and determined that none of the four tools can consistently and accurately diagnose cracked teeth. Regardless of clinical experience, none of the examiners demonstrated a perfect Cohen's kappa score. As demonstrated in Table 1, visual aids have been proven to detect crack location accurately around 50% of the time, compared to micro‐CT.

### 4.1 Diagnostic Accuracy of Index Tests

Although transillumination was the most accurate index test, it was found to have high rates of false positives. Fong et al. (2023) stated that transillumination was the second most commonly used diagnostic tool for crack detection. This study explored the uncertainties Fong et al. (2023) raised regarding the unknown diagnostic accuracy and false positive rate of transillumination. Transillumination resulted in an increase in sensitivity and a decrease in specificity of crack detection. The decrease in specificity is likely due to transillumination's ability to highlight minute physiological craze lines, which can lead to misdiagnosis. As transillumination was performed from six angles for each sample, anatomical features and pathological cracks may have been emphasized, leading to the increased false positive rate. Consequently, clinicians should not solely rely on transillumination (Pitts and Natkin 1983; Sheets et al. 2014).

DIAGNOcam demonstrated the second‐highest accuracy in diagnosing cracks. A recent study reported that due to its high light penetration depth and resolution contrast, DIAGNOcam's ability to verify the presence of a crack is greater than that of fiber‐optic transillumination and microscopy (Hausdörfer et al. 2023). However, their reference standard was ground sections, which are far less accurate than micro‐CT at showing pre‐existing cracks. It is important to note that the light intensity for both DIAGNOcam and transillumination is fixed. Manufacturers should consider allowing the clinician to adjust the light intensity, as the different illumination profiles may improve this tool's visual clarity and usefulness. As previously mentioned, DIAGNOcam emits light from two opposing sources; however, DIAGNOcam does not allow the user to adjust the light intensity or turn a single light off.

High‐magnification microscopy (21.25× end magnification) and macrophotography had high accuracy and PPV. Our study's findings concur with Clark et al.'s report that magnifications greater than 14× aid in accurately diagnosing cracks (Clark et al. 2003). The surgical microscope's higher magnifications (21.25× and 12.5×) were approximately 10% more accurate than the low magnification of 5.1×. This is important in everyday general dental practice, as it seems only 25% of dentists use loupes that are more than 3.5× (Fong et al. 2023).

### 4.2 Significance of Clinical Experience

When the same images were re‐evaluated a few weeks after the initial examination, the examiners agreed with their previous answers only 74%–86% of the time. This highlights that inconsistency is common in image interpretation, as demonstrated in the 70s (Goldman et al. 1972). Concerning inter‐examiner reliability, the significant disagreement between the new graduates (Examiners 3 and 4) is likely due to the variability in clinical exposure. In addition, the undergraduate endodontic curriculum places limited emphasis on diagnosing and managing cracked teeth (Inc 2007; Baaij et al. 2024; Sadr et al. 2021). Further, the criteria of what constitutes a clinically significant crack remained undefined until recently (Lubisich et al. 2010; Hasan et al. 2015). In this context, the recent position statement released by the European Society of Endodontology appears promising (Patel et al. 2025).

### 4.3 Detection of Crack Location

**Figure 6:**
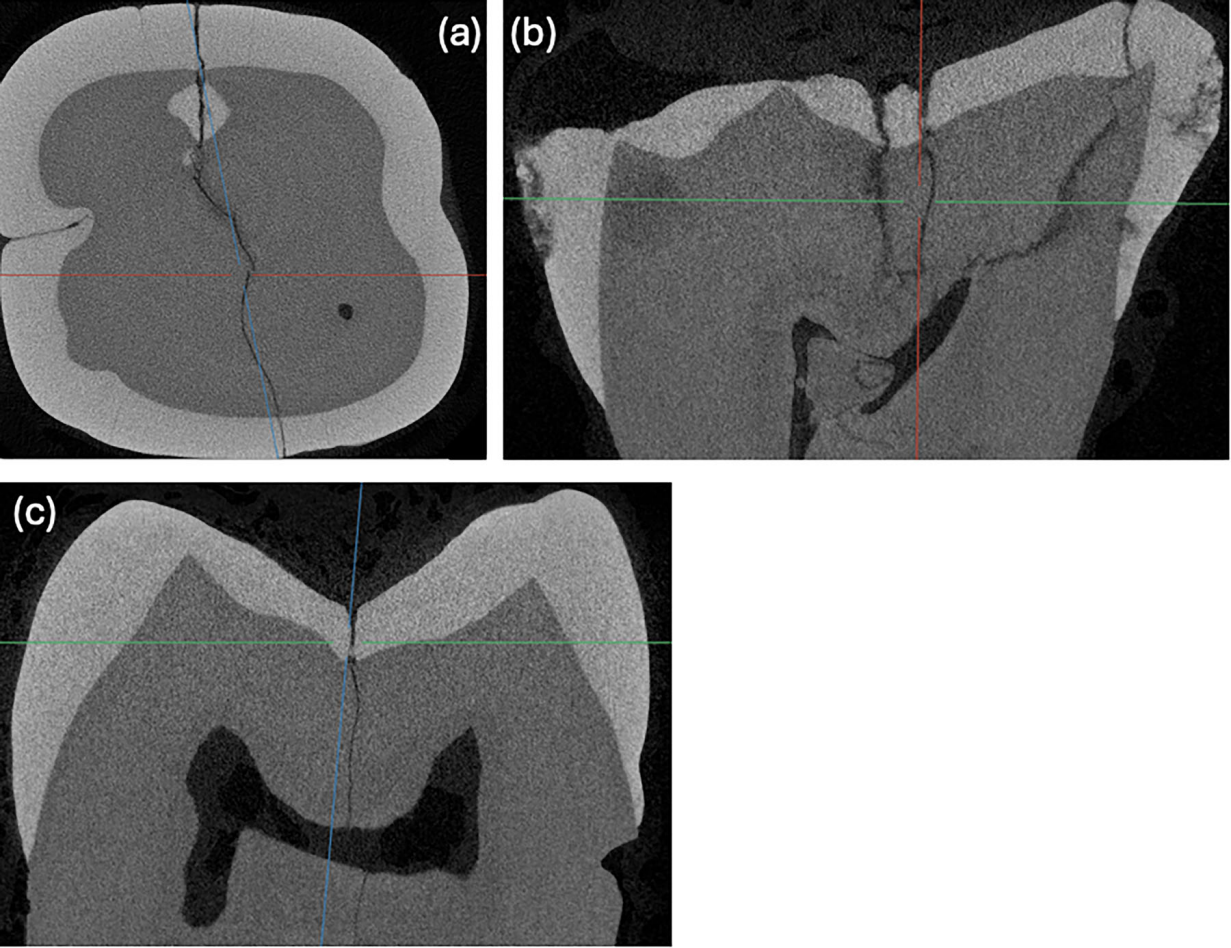
Micro‐CT scans of a sample tooth: (a) axial view, (b) sagittal view, and (c) coronal view.

The complex dentine microstructure and its ability to exhibit crack‐arresting behavior, such as crack blunting, crack bridging, and micro‐cracking, is well established (Kahler et al. 2003; Renson et al. 1974). As demonstrated in Figure 6, crack propagation is nonlinear and multi‐dimensional. This further complicates the ability of a clinician to accurately predict the 3‐dimensional location of the crack. Currently, the available diagnostic tools are vastly inferior at diagnosing locations compared to micro‐CT. None of the tools were beneficial, with only an 11.1% difference in location accuracy between the most accurate tool (transillumination) and the least accurate tool (low‐magnification microscope). Hence, clinicians should not rely on these index tests to detect the precise location of a crack and should consider removing existing restorations (when present) to better visualize the crack location.

### 4.4 Prevalence of Cracked Teeth

**Figure 7:**
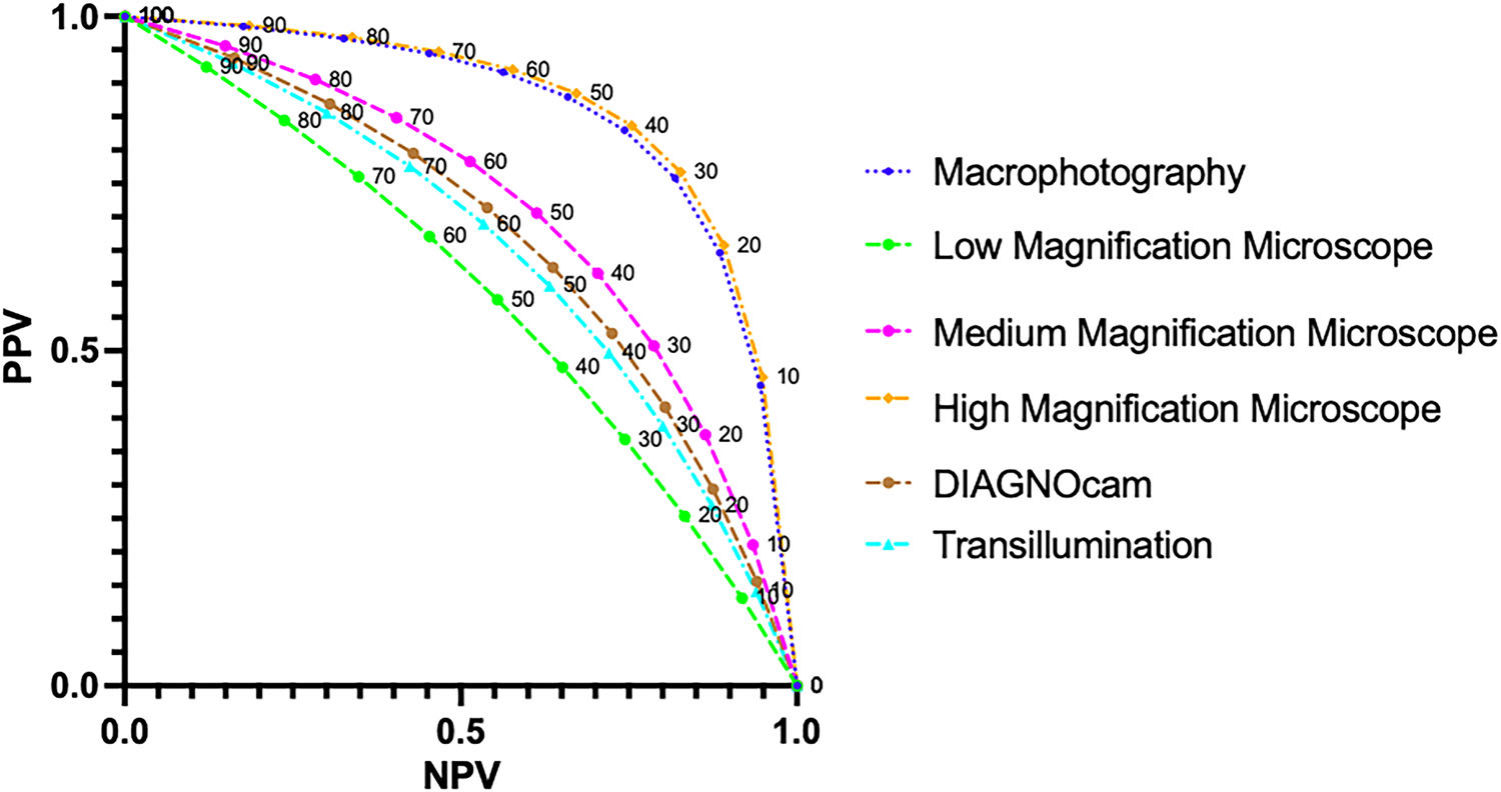
Comparison of diagnostic abilities of four index tests by graphing PPV versus NPV at different prevalences of cracked teeth.

A key contribution of this study was that a model was used to extrapolate the range of possible PPVs and NPVs based on the varying prevalence of cracks (Figure 7). Clinicians should appreciate that both specificity and sensitivity are dependent on prevalence. If the prevalence is 100%, the sensitivity is equal to the accuracy, whereas if the prevalence is 0%, the specificity is equal to the accuracy of the test. Therefore, out of 100 teeth without cracks, the best‐performing test, transillumination, would falsely identify 47 as exhibiting cracks. This could potentially result in overtreatment.

Interestingly, 20% (6 teeth) of the sample did not have crack extension into the DEJ, though they were extracted, suspecting cracked teeth. A separate study will evaluate the clinical parameters and demographics associated with this sample. However, this highlights the difficulties in accurately diagnosing the depth of the crack using currently available methods (Figure 6b).

As evident in Figure 7, a high‐magnification microscope and macrophotography would perform well in both high‐ and low‐prevalence situations. Both at 10% and 70% prevalence, medium‐magnification microscope demonstrates better ability at excluding and confirming crack than transillumination and low‐magnification microscope. In short, the accuracy of the test would change with the prevalence of the condition. Hence, even though transillumination was the most accurate test in this study, a better strategy is to use high magnification or macrophotography as they perform well across high and low prevalence situations.

The questions of overdiagnosis and overtreatment in relation to cracked teeth are significant. Among the 2601 teeth investigated in a recent National practice‐based research network study on cracked teeth, over a 3‐year period, only 3% of all teeth identified as cracked developed a fracture (Hilton et al. 2021). Additionally, out of the 1889 untreated teeth, only 12% showed crack progression (Hilton et al. 2021). The generally slow rate of crack advancement and low incidence of complications such as tooth fracture, coupled with the inaccuracies of available diagnostic tools, should encourage clinicians to consider minimally invasive strategies in managing cracked teeth (Hilton et al. 2021).

## 5 Conclusion

This study highlighted the deficiencies of visual aids in establishing accurate and reproducible crack diagnosis when used in isolation. Future research should focus on using a combination of diagnostic tools to detect cracks. A clinically relevant diagnostic strategy for cracked teeth diagnosis must be developed to manage the uncertainty in this space.

## Author Contributions

All authors have contributed substantially to conception and design, acquisition of data, or analysis and interpretation of data; been involved in drafting the manuscript or revising it critically for important intellectual content; given final approval of the version to be published. Each author should have participated sufficiently in the work to take public responsibility for appropriate portions of the content and agreed to be accountable for all aspects of the work in ensuring that questions related to the accuracy or integrity of any part of the work are appropriately investigated and resolved.

## Conflicts of Interest

The authors declare no conflicts of interest.

## Supporting information

Supporting file 1 CDR.

## Data Availability

The data that support the findings of this study are available from the corresponding author upon reasonable request.
