# Supplementary material for: Diagnostic Accuracy of Methods Used to Detect Cracked Teeth
Source: Clin Exp Dent Res. 2025 Apr 30;11(3):e70138. doi: 10.1002/cre2.70138 (PMC12042108; doi:10.1002/cre2.70138)

## Slide 1
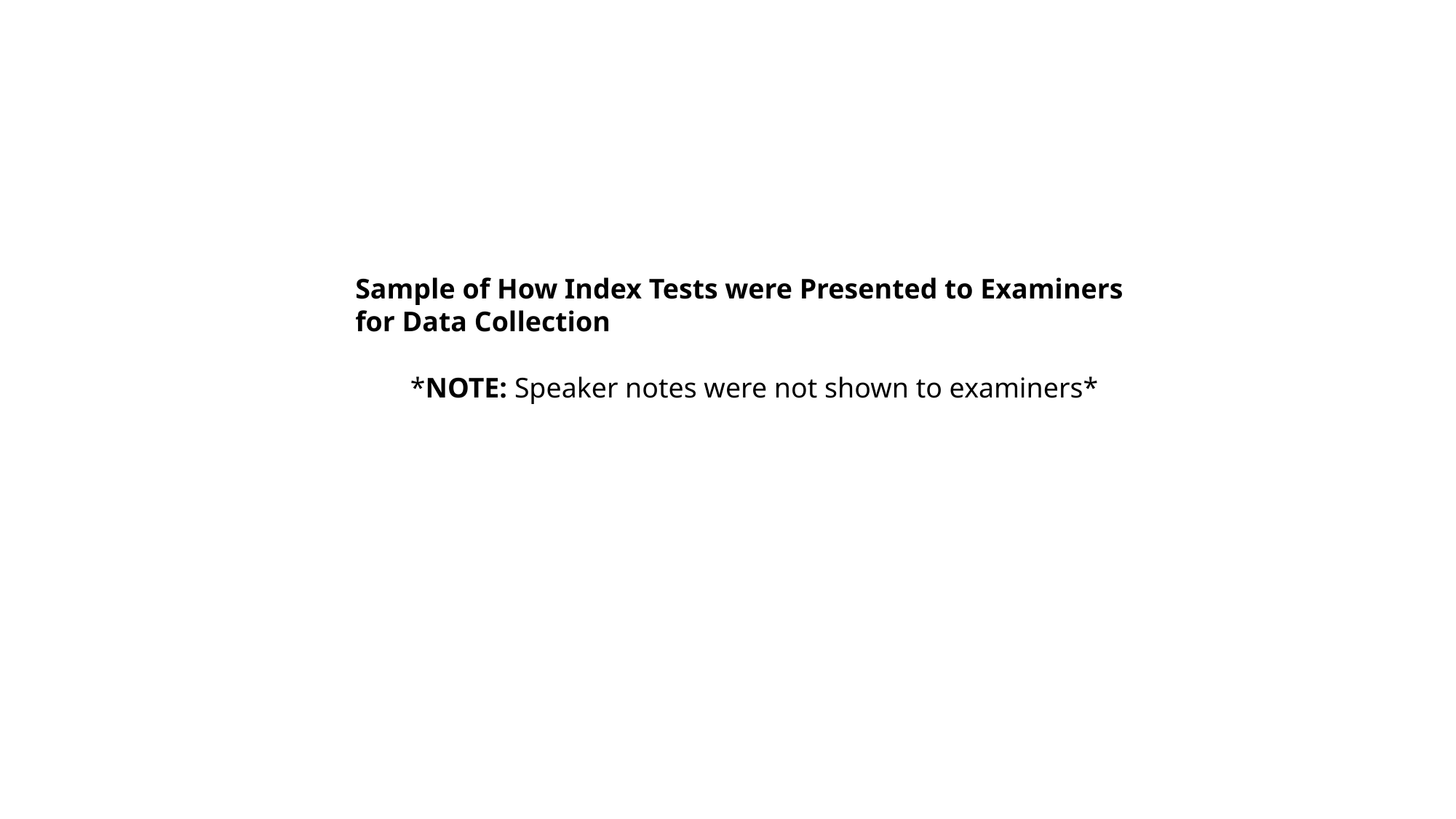

Sample of How Index Tests were Presented to Examiners for Data Collection
*NOTE: Speaker notes were not shown to examiners*

## Slide 2
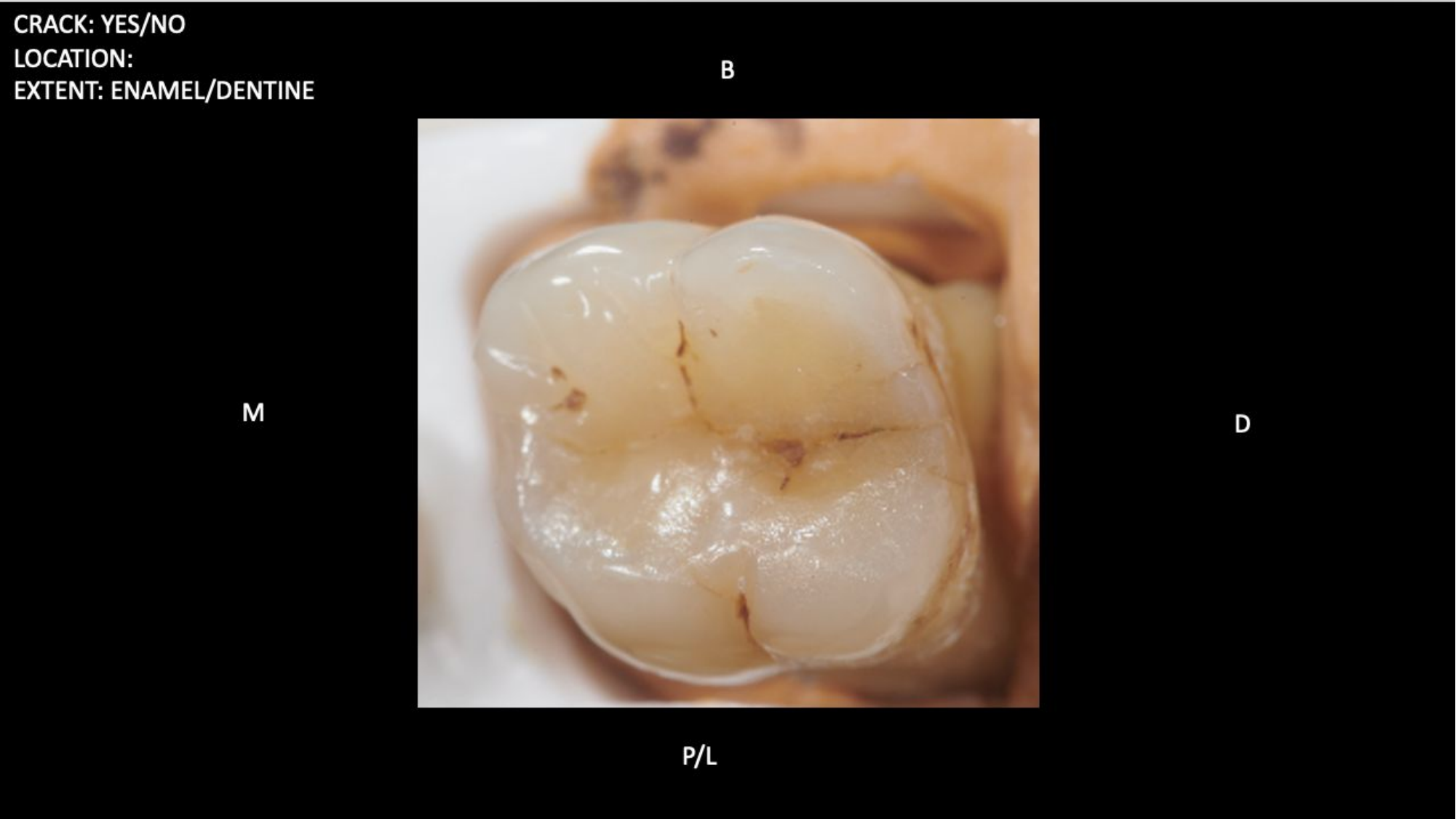

## Slide 3
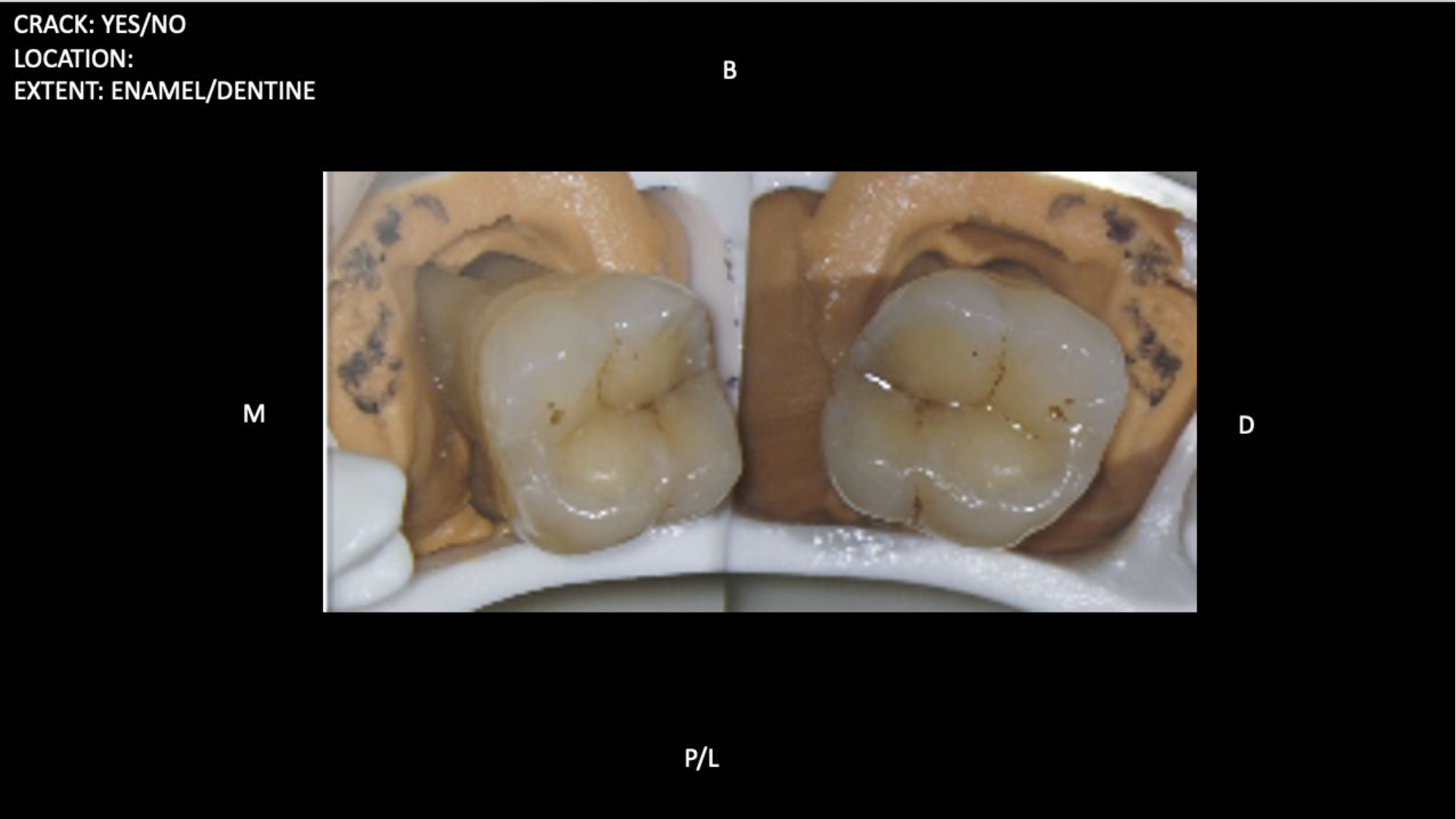

## Slide 4
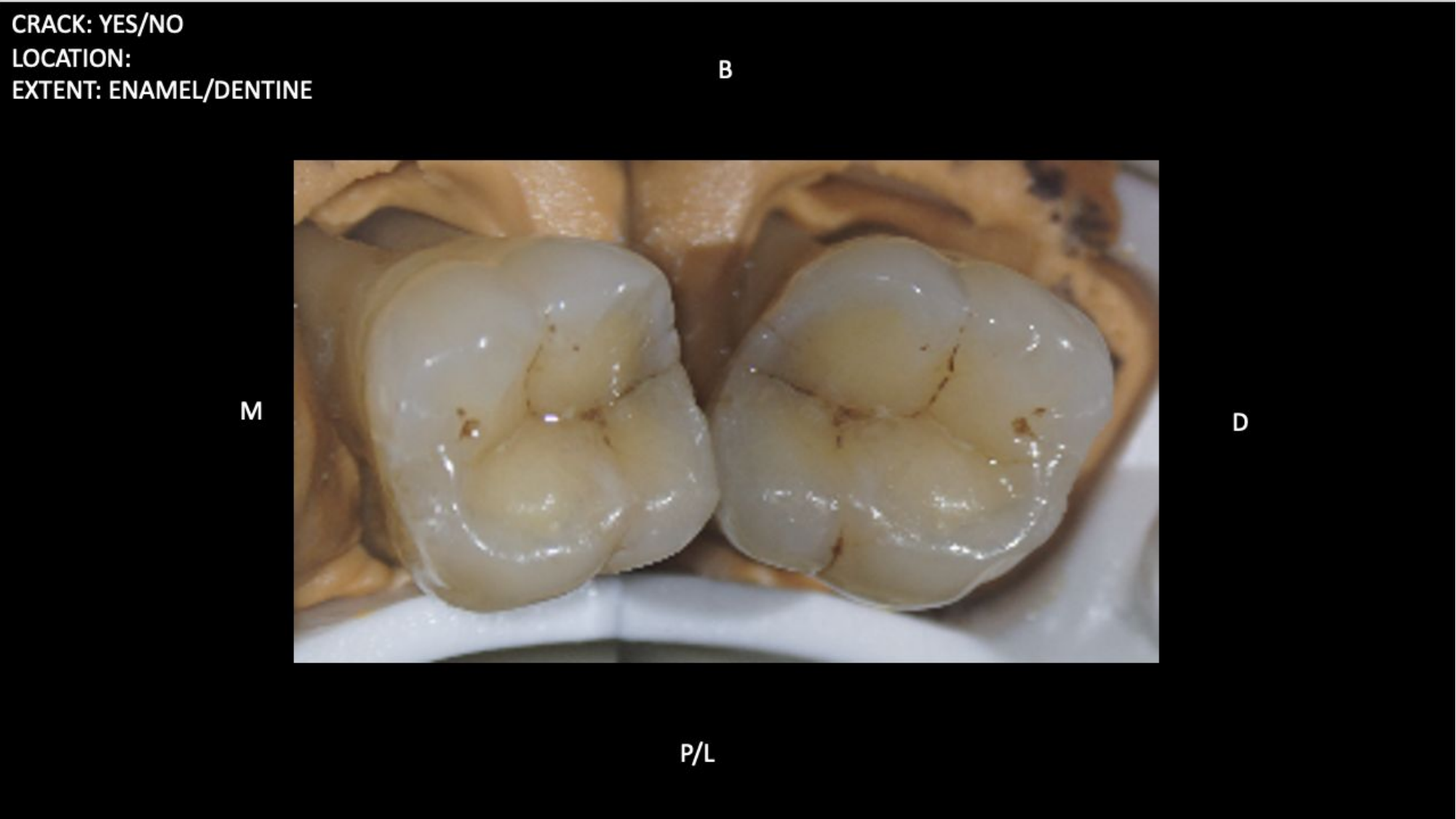

## Slide 5
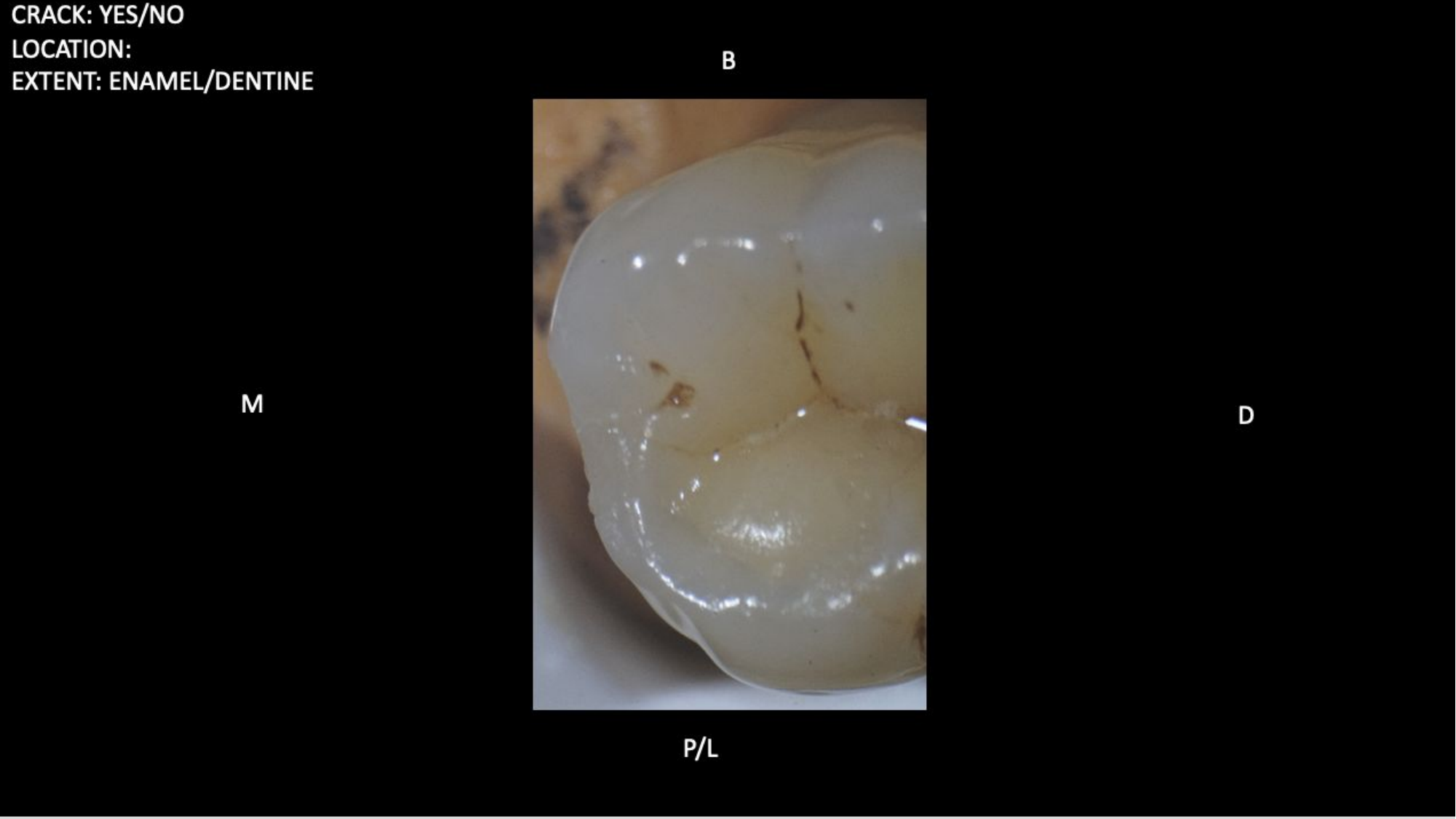

## Slide 6
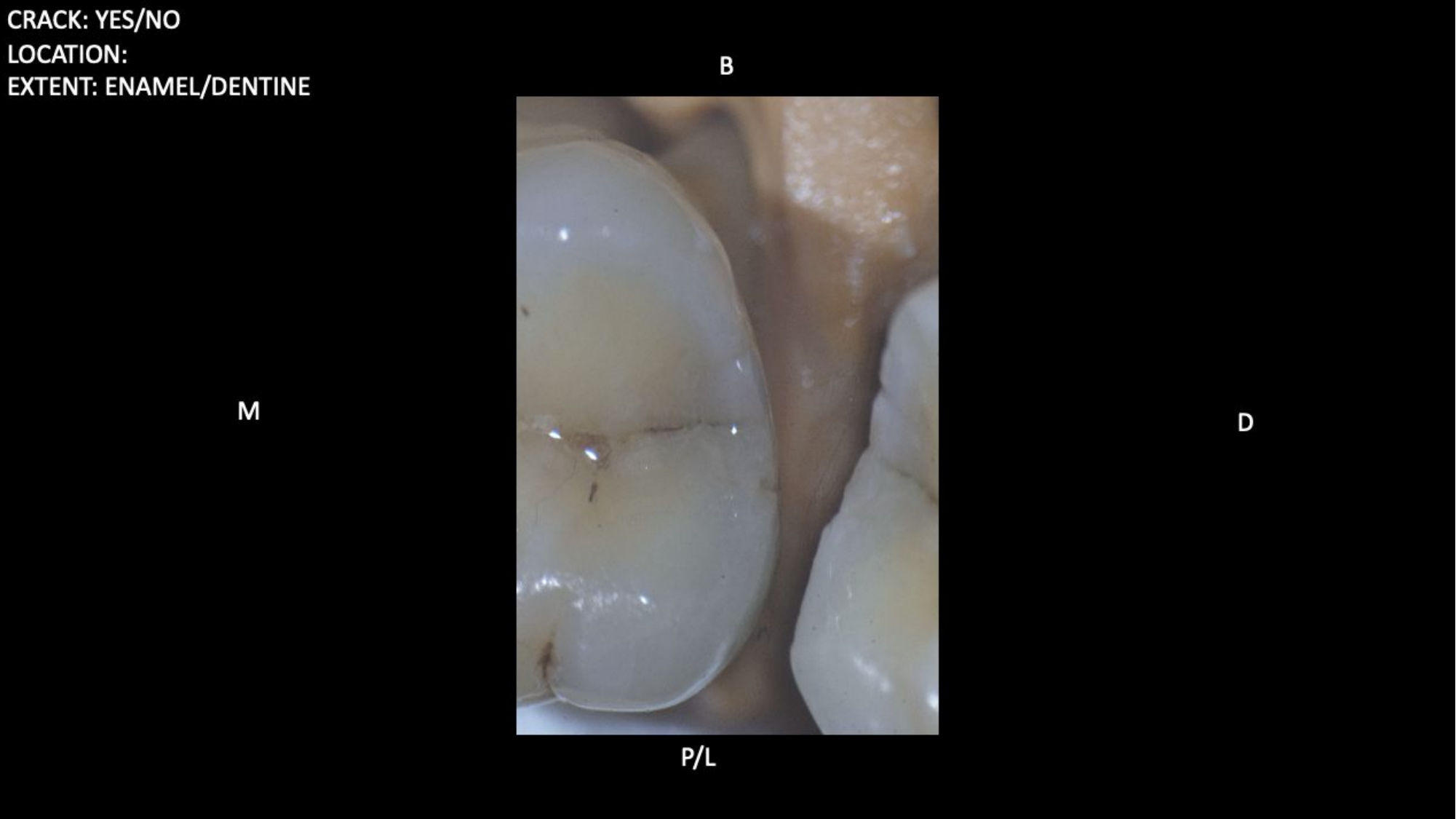

## Slide 7
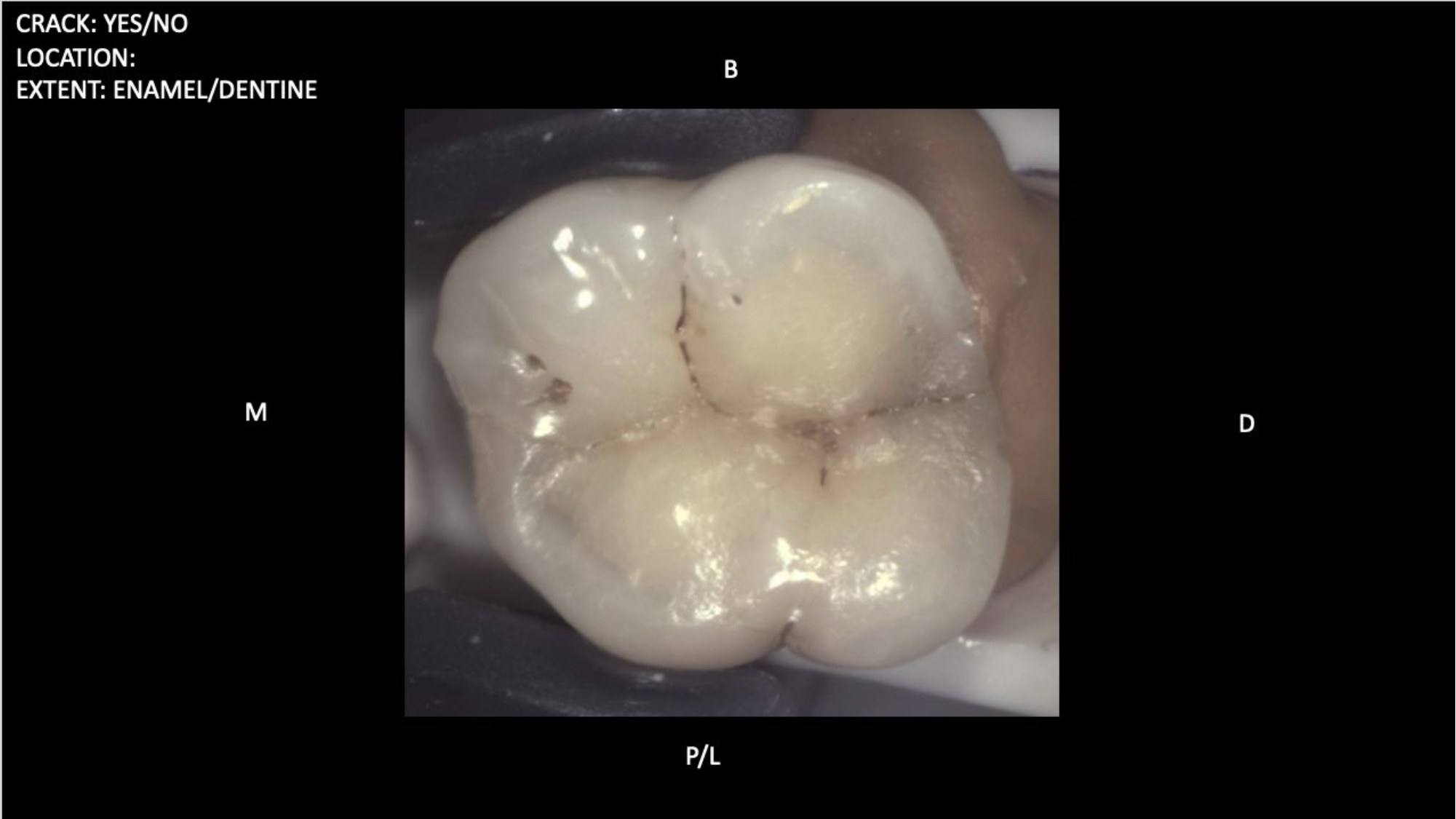

## Slide 8
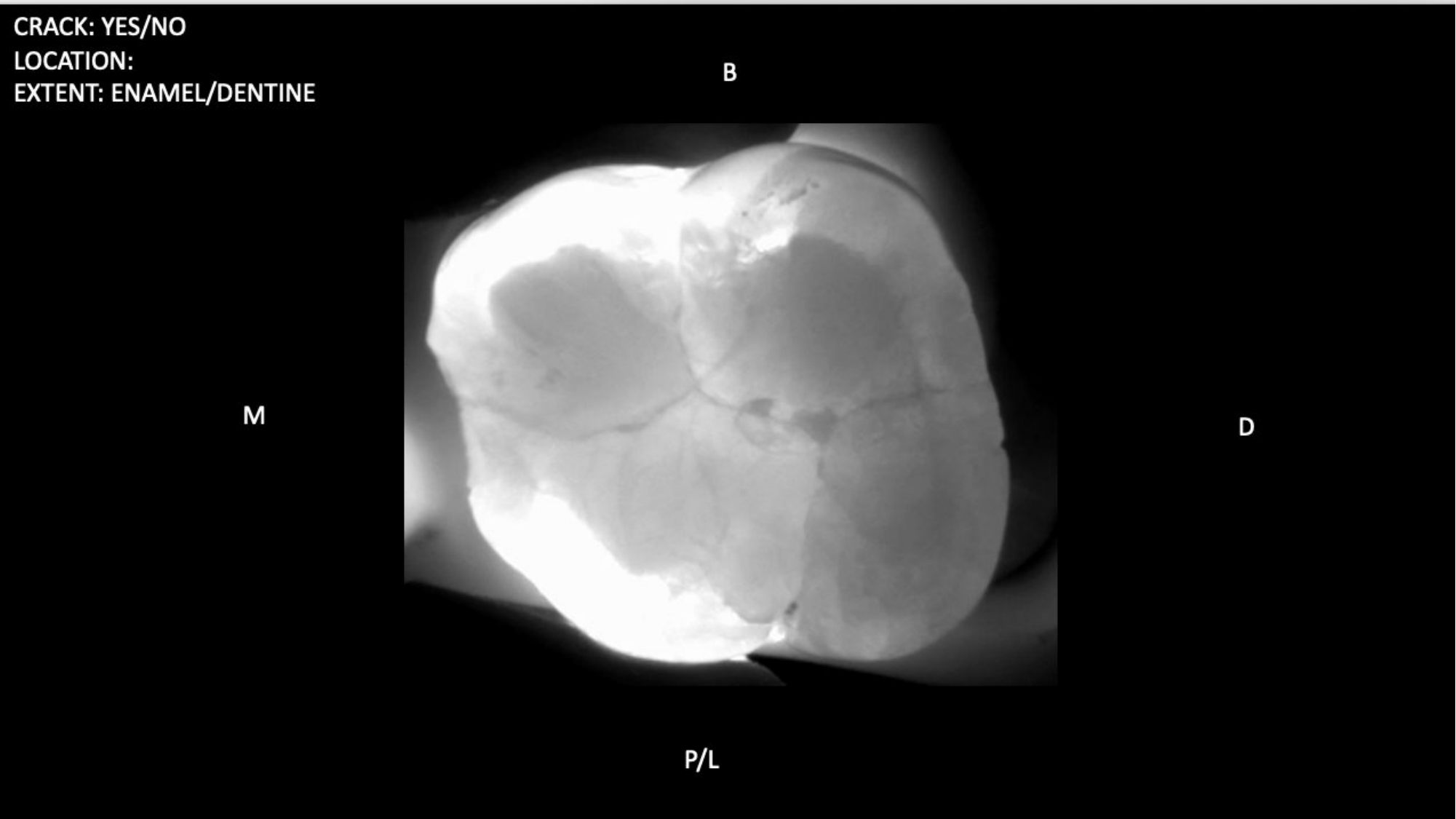

## Slide 9
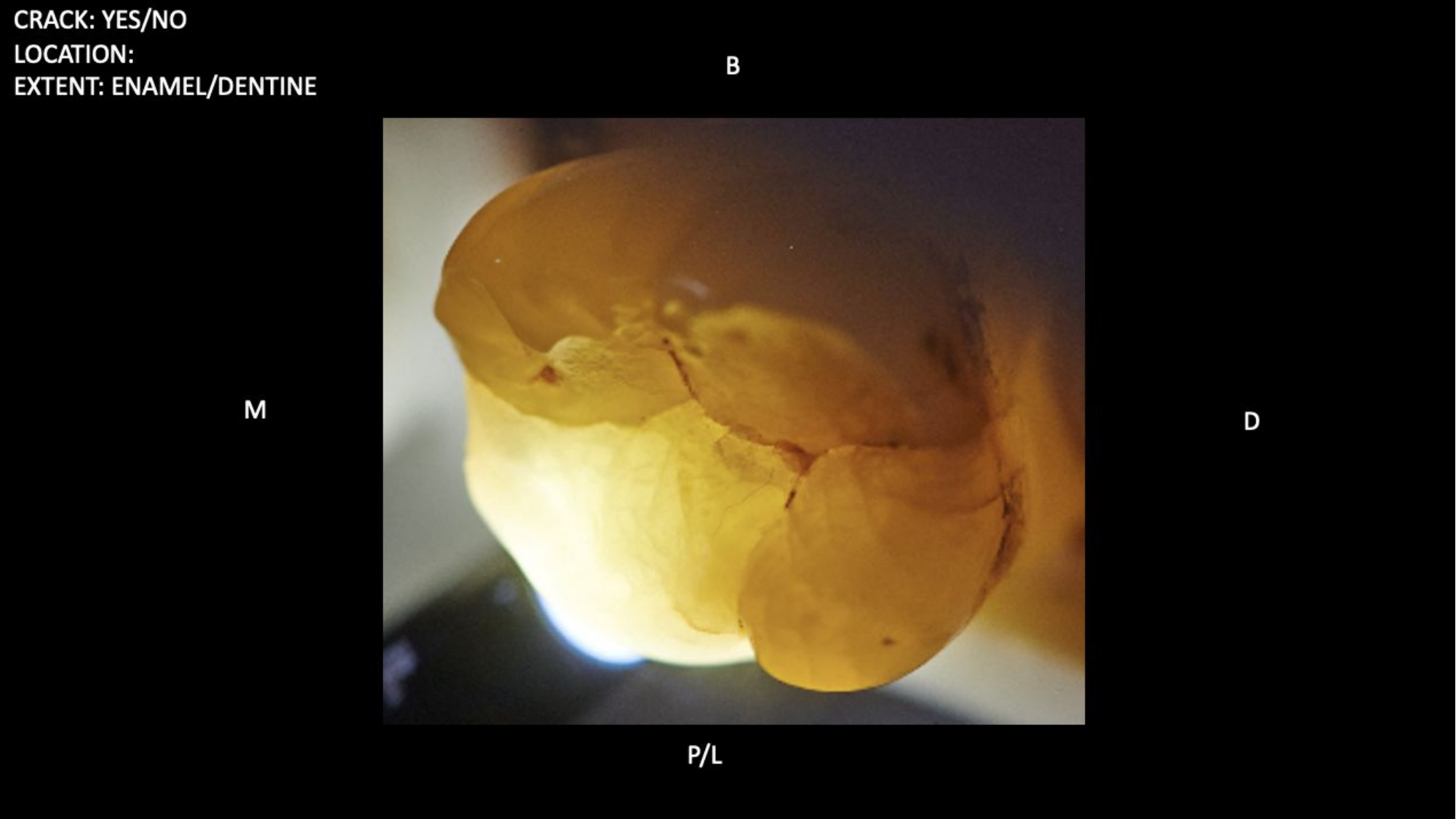

## Slide 10
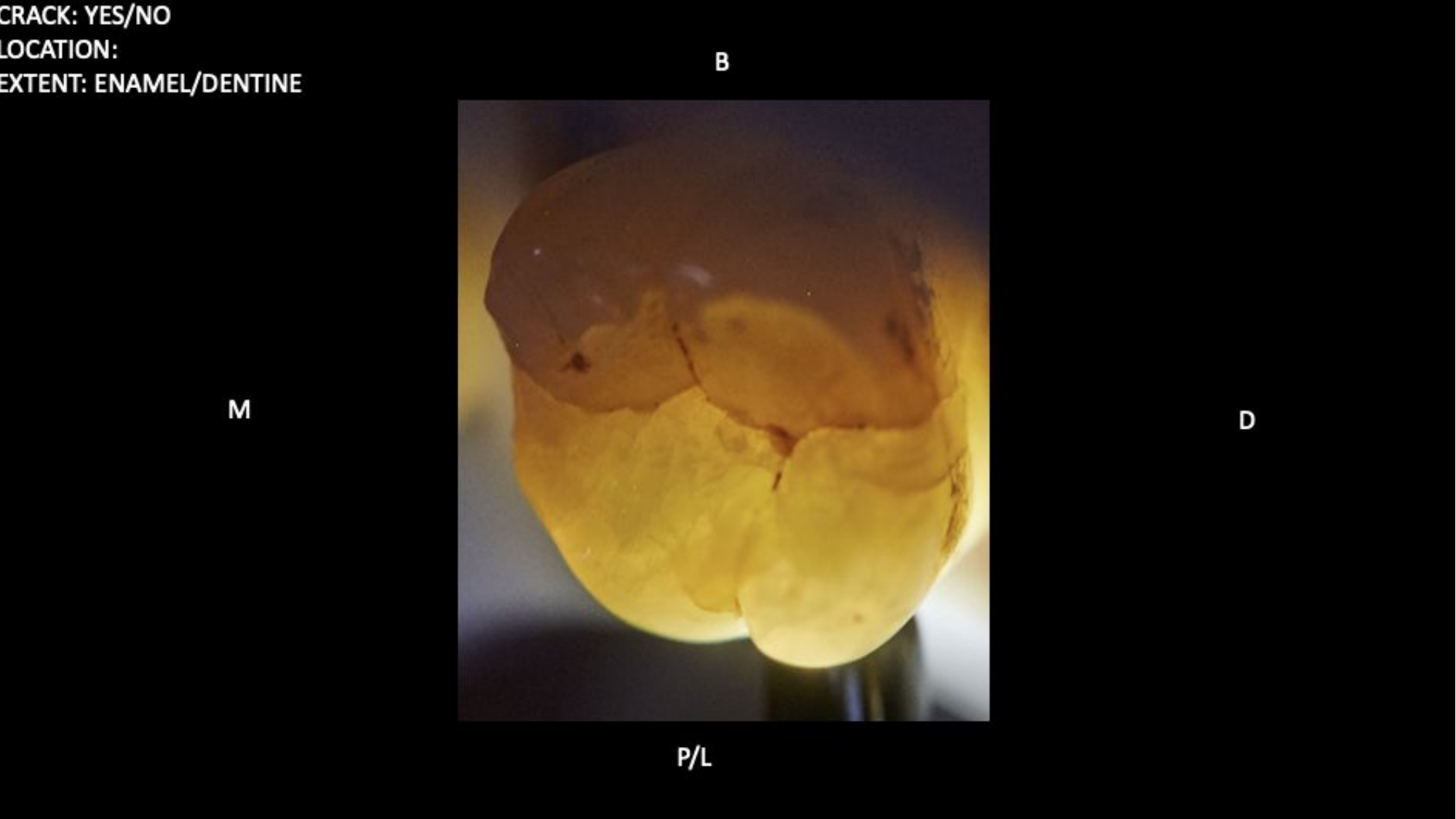

## Slide 11
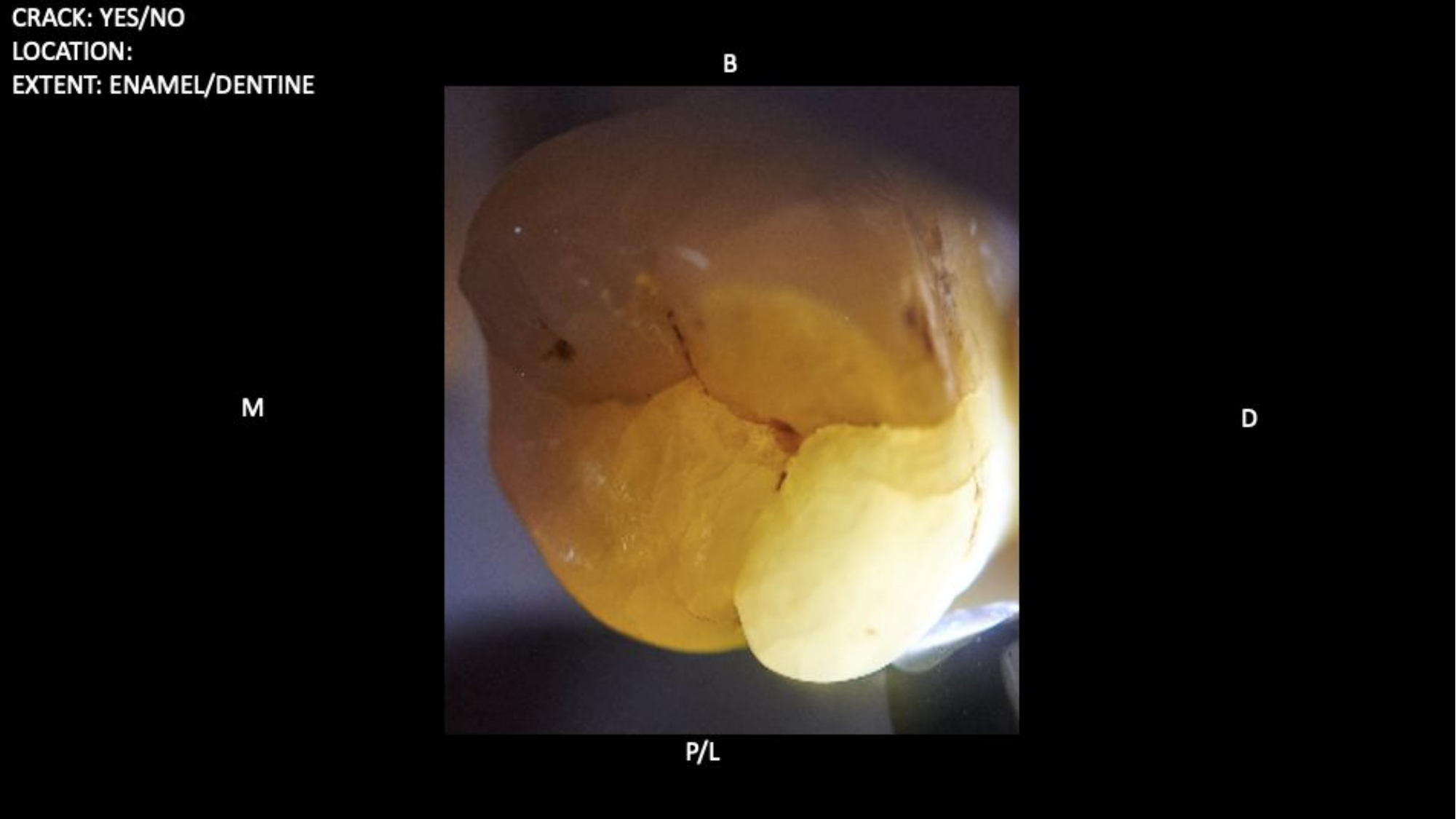

## Slide 12
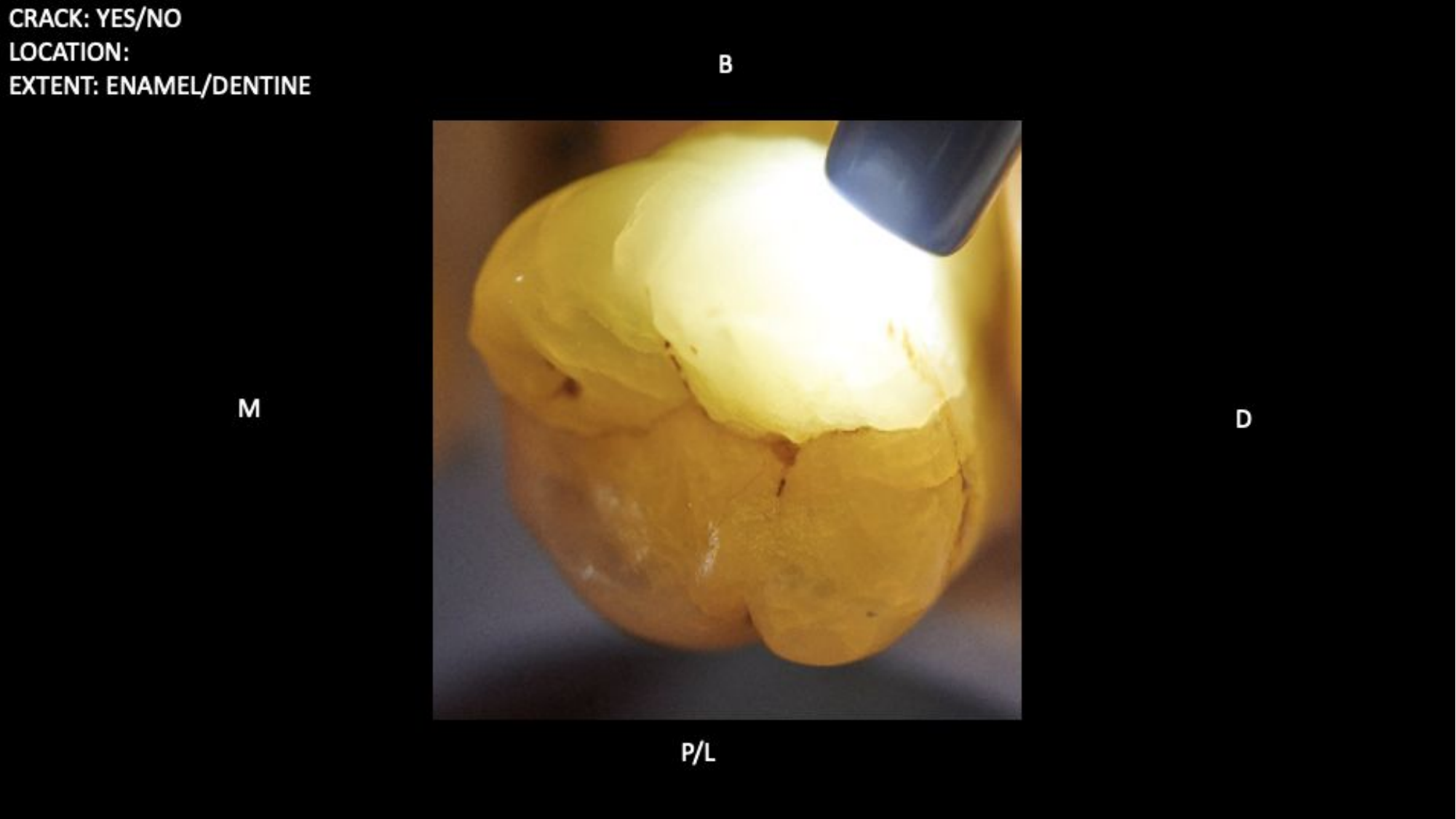

## Slide 13
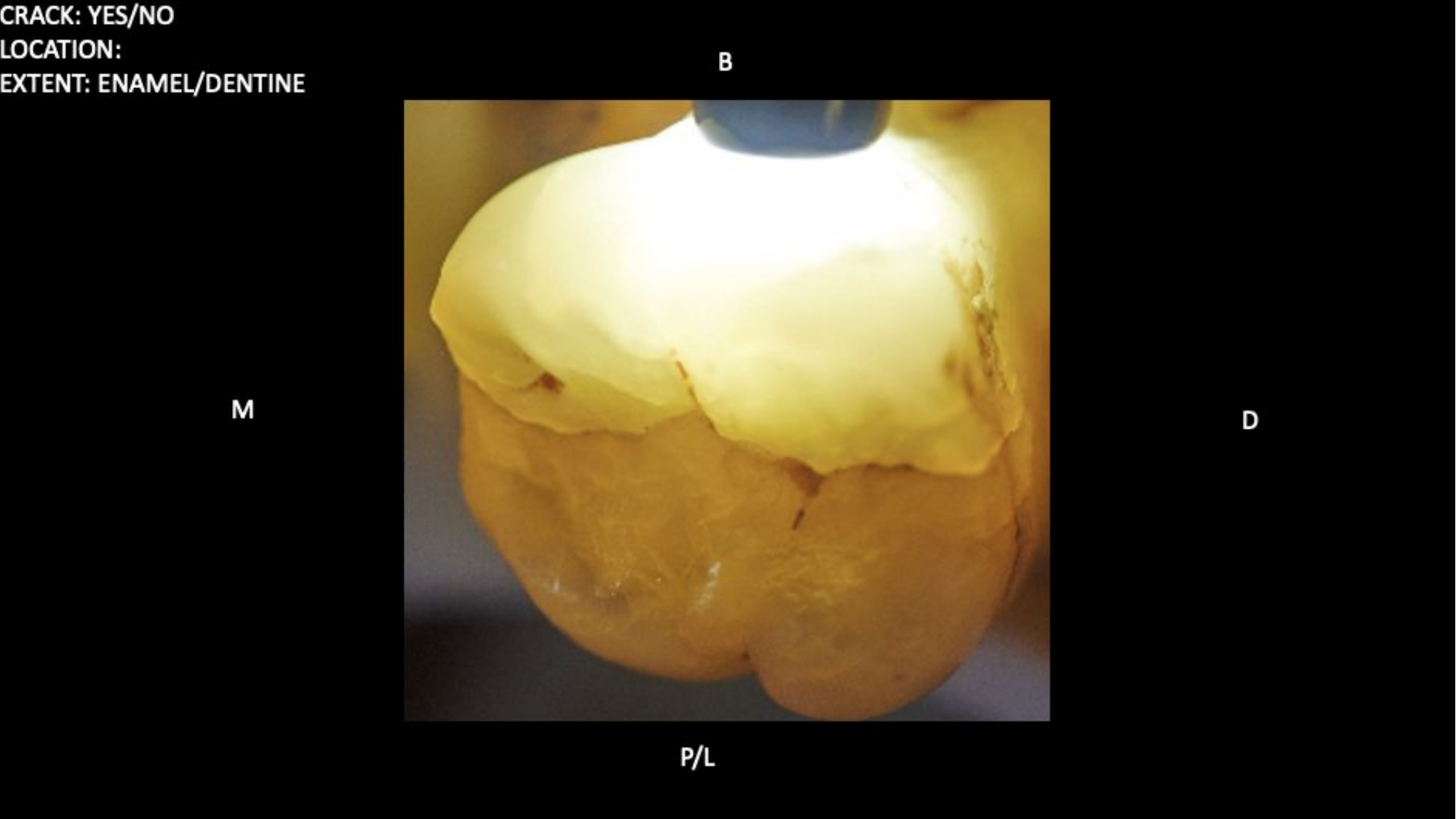

## Slide 14
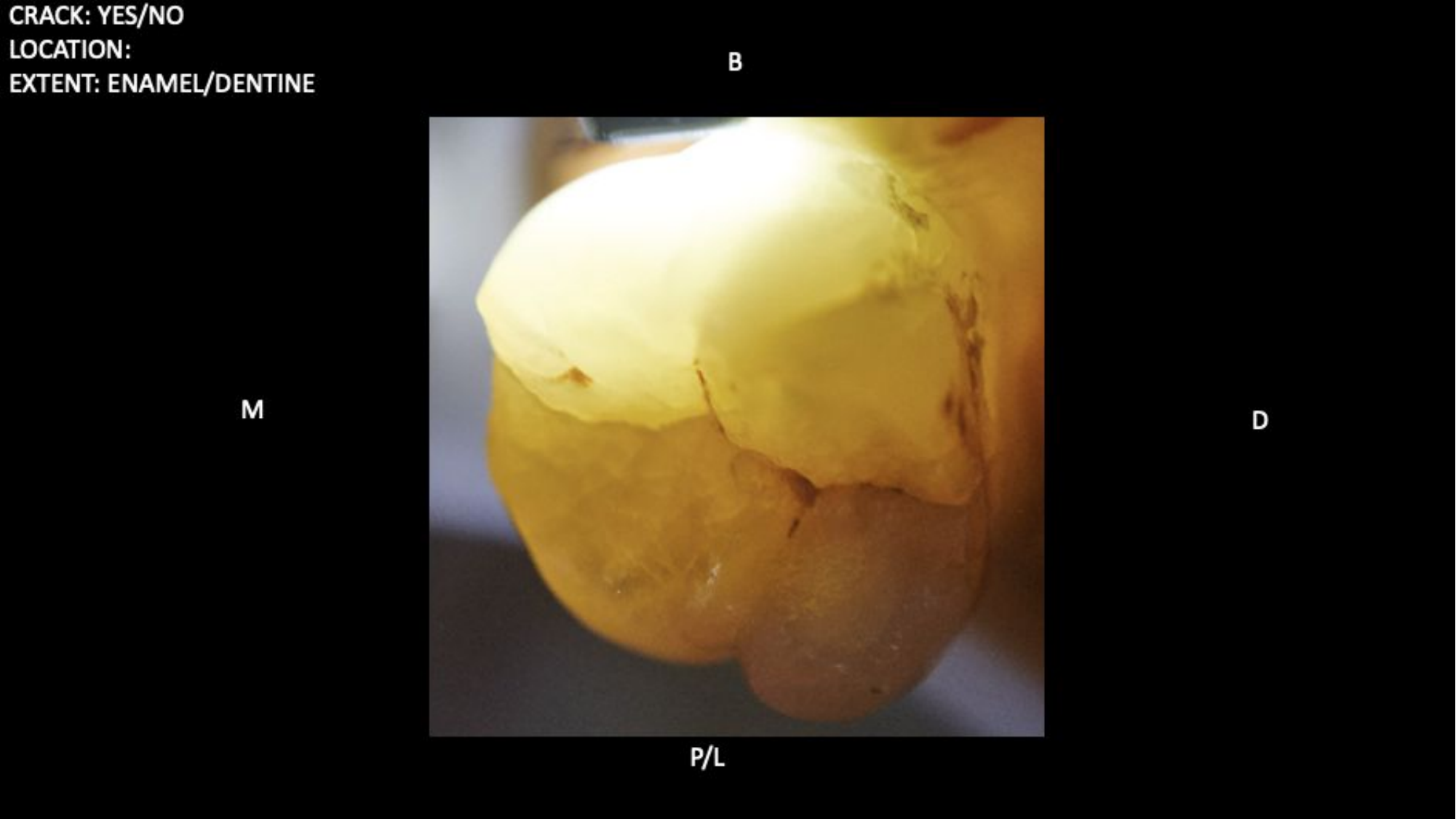

Supplement: Supplementary file 1 — Supporting file 1 CDR. [file CRE2-11-e70138-s001.pptx]
